# Emerging Novel Reassortant Influenza A(H5N6) Viruses in Poultry and Humans, China, 2021

**DOI:** 10.3201/eid2805.212163

**Published:** 2022-05

**Authors:** Wenming Jiang, Chunxia Dong, Shuo Liu, Cheng Peng, Xin Yin, Shaobo Liang, Lin Zhang, Jinping Li, Xiaohui Yu, Yang Li, Jingjing Wang, Guangyu Hou, Zheng Zeng, Hualei Liu

**Affiliations:** China Animal Health and Epidemiology Center, Qingdao, China (W. Jiang, S. Liu, C. Peng, X. Yin, S. Liang, L. Zhang, J. Li, X. Yu, Y. Li, J. Wang, G. Hou, H. Liu);; Chongqing Animal Disease Prevention and Control Center, Chongqing, China (C. Dong, Z. Zeng)

**Keywords:** influenza, avian influenza, Novel H5N6 virus, highly pathogenic, vaccination, antigenic characterization, viruses, respiratory infections, zoonoses, vaccine-preventable diseases, China

## Abstract

A novel highly pathogenic avian influenza A(H5N6) clade 2.3.4.4b virus was isolated from a poultry market in China that a person with a confirmed case had visited. Most genes of the avian and human H5N6 isolates were closely related. The virus also exhibited distinct antigenicity to the Re-11 vaccine strain.

Highly pathogenic avian influenza A(H5N1) virus emerged in China in 1996. H5 viruses have spread to Eurasia since 2003, Africa since 2005, and North America since 2014–2015. These viruses cause huge economic losses to the poultry industry and pose substantial threats to human health. By March 2022, a total of 75 confirmed cases of human infection with influenza A(H5N6) virus had been reported, including 48 cases in China since 2021 (https://www.who.int/teams/global-influenza-programme/avian-influenza/monthly-risk-assessment-summary).

On July 9, 2021, a human case of H5N6 infection was reported in Chongqing, China. One day later, we conducted an epidemiologic survey in the poultry market the patient had visited and collected swab samples from poultry. We identified the samples as H5N6 subtype by using H5- and N6-specific primers and probes. We propagated the virus in 10-day-old specific pathogen–free chicken embryos and designated the isolate as A/chicken/Chonqqing/H1/2021(H5N6) (CK/CQ/H1). We sequenced the viral genome by using the Sanger method and deposited the sequences in GISAID (https://www.gisaid.org; accession nos. EPI1937512–9).

Phylogenetic analysis of the hemagglutinin (HA) genes showed that CK/CQ/H1 and A/Chongqing/02/2021 were closely related genetically and belonged to subclade 2.3.4.4b, along with H5N6 human isolates from Sichuan (2021) and Hunan (2021) Provinces, indicating that their HA genes likely derived from wild bird strains that arrived in China in 2020 (Figure). Phylogenetic analysis of the neuraminidase (NA) genes showed that the isolate were most closely related to H5N6 isolates (subclade 2.3.4.4h) from China (Figure). The H5N8 viruses that arrived in China in late 2020 appear to have reassorted with clade 2.3.4.4h H5N6 viruses already circulating.

**Figure:**
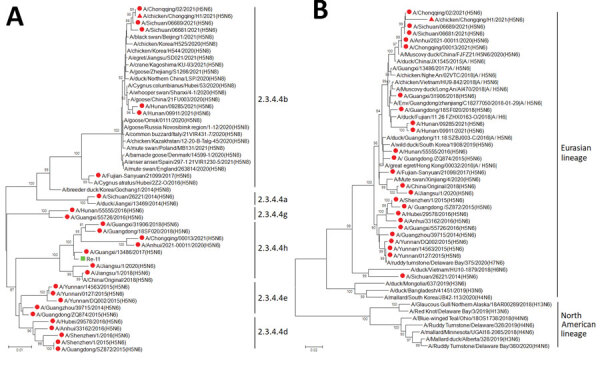
Phylogenetic trees of hemagglutinin (A) and neuraminidase (B) genes of H5 and N6 subtype influenza viruses collected from poultry and humans in China, 2021, and reference viruses. Red triangles indicate virus obtained in this study; red circles indicate human-infected avian influenza viruses; green squares indicate H5 Re-11 vaccine strain. Clade numbers and lineages are indicated on the right in panel. Trees were constructed with MEGA 5.10 software (https://www.megasoftware.net) using the neighbor-joining method. Bootstrap analysis was performed with 1,000 replications. Scale bars indicate nucleotide substitutions per site.

Sequence analysis suggested that the polymerase basic protein 1, polymerase acidic protein, and nucleoprotein genes of CK/CQ/H1 were closely related to those of H5N6 viruses in China, such as A/Environment/Guangdong/C18277136/2018(H5N6) and A/Muscovy duck/China/FJFZ21/2020(H5N6). The matrix protein gene was most closely related to those of H5N8 viruses in Korea and China such as A/wild bird/Korea/H496–3/2020(H5N8) and A/Cygnus columbianus/Hubei/49/2020(H5N8), the polymerase basic protein 2 gene to those of A/Environment/Guangxi/28753/2014(H3N2), and the nonstructural protein gene to those of A/Environment/Jiangxi/47054/2016(H4N2) (Appendix Table 1). These findings indicate that CK/CQ/H1 is a new reassortant virus with genes derived from different avian influenza virus subtypes in eastern Asia.

Analysis based on the HA amino acid sequence revealed the presence of a cleavage site (PLREKRRKR/GLF), suggesting that the isolate was highly pathogenic in chickens. The presence of receptor binding sites Q226 and G228 (H3 numbering) indicate that the isolate would preferentially bind to avian-like receptors (*1*). However, the receptor binding site mutations A137, N158, A160, N186, I192, Q222, and R227 (H3 numbering) could increase binding to human-like receptors (*2*–*5*).

Bioinformatics analysis identified many mutations that would increase virulence in mice, such as R114 and I115 (H3 numbering) of the HA gene; D30, M43, and A215 of the matrix protein 1 gene; S42, E55E, E66, M106, and F138 of the nonstructural protein 1 gene; the nonstructural protein 1 C-terminal ESEV motif of the PDZ domain at position aa 227–230; V89, D309, K339, G477, V495, E627, and T676 of the polymerase basic protein 2 gene; V3 and G622 of the polymerase basic protein 1 gene; and D383 of the polymerase acidic protein gene (*6*). Mice inoculated with CK/CQ/H1 experienced a rapid and dramatic weight loss of >30%, had signs of illness, and died within 8 days (Appendix Figure).

Since 2019, the inactivated reassortant vaccine H5 Re-11 (clade 2.3.4.4h) has been used in China to control clade 2.3.4.4 viruses. We analyzed differences in antigenicity between CK/CQ/H1 and Re-11. The hemagglutination inhibition titer of Re-11 antiserum against CK/CQ/H1 was 5 log_2_ lower than that against the homologous Re-11 antigen, indicating that CK/CQ/H1 exhibited greater antigenic drift relative to the Re-11 vaccine strain. The variations of antigenicity-associated amino acid sites on HA might indicate the potential antigenic drift of CK/CQ/H1 (*7*) (Appendix Table 2).

We also evaluated the protective efficiency of Re-11 vaccine against the isolate. We vaccinated 3-week-old specific pathogen–free chickens with the Re-11 vaccine. At 21 days after vaccination, the vaccine induced very high levels of antibody against the vaccine antigen. Then, the birds were intranasally challenged with 10^6^ 50% egg infectious dose of CK/CQ/H1. All vaccinated birds displayed no clinical signs and survived, but 2 of them shed virus (Appendix Table 3). The results were inconsistent with those of Cui et al. (*8*), which may be related to bird species and immune background.

Novel H5N8 viruses of clade 2.3.4.4b virus have spread to China through migratory birds in late 2020 (*9*,*10*). These viruses are similar to those that were dominant in Europe from the autumn of 2020 through 2021 but have undergone reassortment since arriving in China, producing novel viruses like CK/CQ/H1. The novel virus we identified is highly pathogenic to both chickens and mice and exhibited distinct antigenicity to the Re-11 vaccine strain, which could not provide complete protection. Under field conditions, birds are unlikely to get sustained high levels of antibody and would more likely be susceptible to infection and virus shedding. New antigen-matched vaccines and more productive measures are needed to prevent and control novel H5N6 infection in poultry and humans.

Appendix Additional information about emerging novel reassortant influenza A(H5N6) viruses in poultry and humans, China, 2021.
