# Supplementary material for: Emerging Novel Reassortant Influenza A(H5N6) Viruses in Poultry and Humans, China, 2021
Source: Emerg Infect Dis. 2022 May;28(5):1064–6. doi: 10.3201/eid2805.212163 (PMC9045449; doi:10.3201/eid2805.212163)
Supplement: Appendix — Additional information about emerging novel reassortant influenza A(H5N6) viruses in poultry and humans, China, 2021. [file 21-2163-Techapp-s1.pdf]

Article DOI: https://doi.org/10.3201/eid2805.212163

Emerging Novel Reassortant Influenza A(H5N6) Viruses in Poultry and Humans, China, 2021

Appendix

Appendix Table 1. Percentage homology of influenza viruses closely related to A/chicken/Chongqing/H1/2021(H5N6).

Segment	Virus	Nucleotide homology (%)
PB2	A/Sichuan/06681/2021(H5N6)	99.4
	A/Environment/Guangxi/28753/2014(H3N2)	97.3
PB1	A/Sichuan/06681/2021(A/H5N6)	98.5
	A/Environment/Guangdong/C18277136/2018(H5N6)	97.8
PA	A/Sichuan/06681/2021(A/H5N6)	99.7
	A/Environment/Guangdong/C17277346/2017(H5N6)	97.6
HA	A/Chonqqing/02/2021(H5N6)	99.9
	A/Sichuan/06689/2021(H5N6)	99.8
	A/chicken/Korea/H544/2020(H5N8)	99.6
	A/duck/Korea/H538/2020(H5N8)	99.6
	A/duck/Korea/H499/2020(H5N8)	99.6
NP	A/Sichuan/06681/2021(H5N6)	99.6
	A/Anhui/2021–00011/2020(H5N6)	99.3
	A/Muscovy duck/China/FJFZ21/2020(H5N6)	99.3
NA	A/Chonqqing/02/2021(H5N6)	97.5
	A/Sichuan/06689/2021(H5N6)	97.5
	A/Sichuan/06681/2021(H5N6)	97.4
	A/Anhui/2021–00011/2020(H5N6)	97.1
	A/Chongqing/00013/2021(H5N6)	97.0
	A/Muscovy duck/China/FJFZ21/2020(H5N6)	96.6
M	A/Cygnus columbianus/Hubei/49/2020(H5N8)	99.8
	A/wild bird/Korea/H496–3/2020(H5N8)	99.8
	A/chicken/Kostroma/304–10/2020 (H5N8)	99.8
	A/mallard/Kagoshima/KU-d89/2021 (H5N8)	99.8
	A/duck/Northern China/LSP/2020(H5N8)	99.8
	A/turkey/Omsk/0003/2020(H5N8)	99.8

Segment	Virus	Nucleotide homology (%)
NS	A/Sichuan/06681/2021(H5N6)	99.6
	A/Environment/Jiangxi/47054/2016(H4N2)	98.0
	A/Environment/Hunan/34019/2017(H3N2)	97.9

Appendix Table 2. The differences in antigenicity sites on HA between A/chicken/Chongqing/H1/2021(H5N6) and Re-11 vaccine strain

Strains	site A					site B			site C	site D				site E		Other antigenicity associated sites in HA1 (except regions A, B, C, D, E)									
H3 numbering	130	133	144	145	128	155	187	192	276	173	227	238	240	260	262	93	101	119	120	125	166	202	271	272	285
Re-11	/	S	V	A	S	T	S	T	N	K	H	R	N	I	T	S	N	T	Q	R	M	V	E	I	I
CK/CQ/H1	E	L	A	P	P	I	N	I	H	R	R	K	D	V	K	A	S	I	L	S	I	I	G	V	V

Appendix Table 3. Protection efficacy of H5 Re-11 vaccine against A/chicken/Chongqing/H1/2021(H5N6) in chickens*

				Challenge test results, by swab type, no. positive birds/no.tested (mean titer±SD)†				
		HI titer±SD, log2		3 dpi		5 dpi		No.surviving
Group	Vaccine	Re-11	Isolate	Tracheal	Cloacal	Tracheal	Cloacal	birds/total no.
CK/CQ/H1	Re-11	9.3 ± 0.38	4.2 ± 0.32	2/10 (3.67 ± 1.18)	2/10 (3.81 ± 1.32)	2/10 (3.79 ± 1.26)	2/10 (3.92 ± 1.42)	10/10
Mock		ND	ND	4/4 (4.10 ± 1.23)	4/4 (4.63 ± 1.76)	ND	ND	0/10

*Chickens were immunized with the Re-11 vaccine, and HI antibody titers were determined on day 21 post-vaccination. HI, hemagglutination inhibition assay; dpi, days post-infection; ND, not done.

†Chickens were challenged with 10^6 50% egg infectious dose (EID_{50}) of A/chicken/Chongqing/H1/2021(H5N6) virus; virus titers are expressed as log10 EID_{50}/0.1 ml.

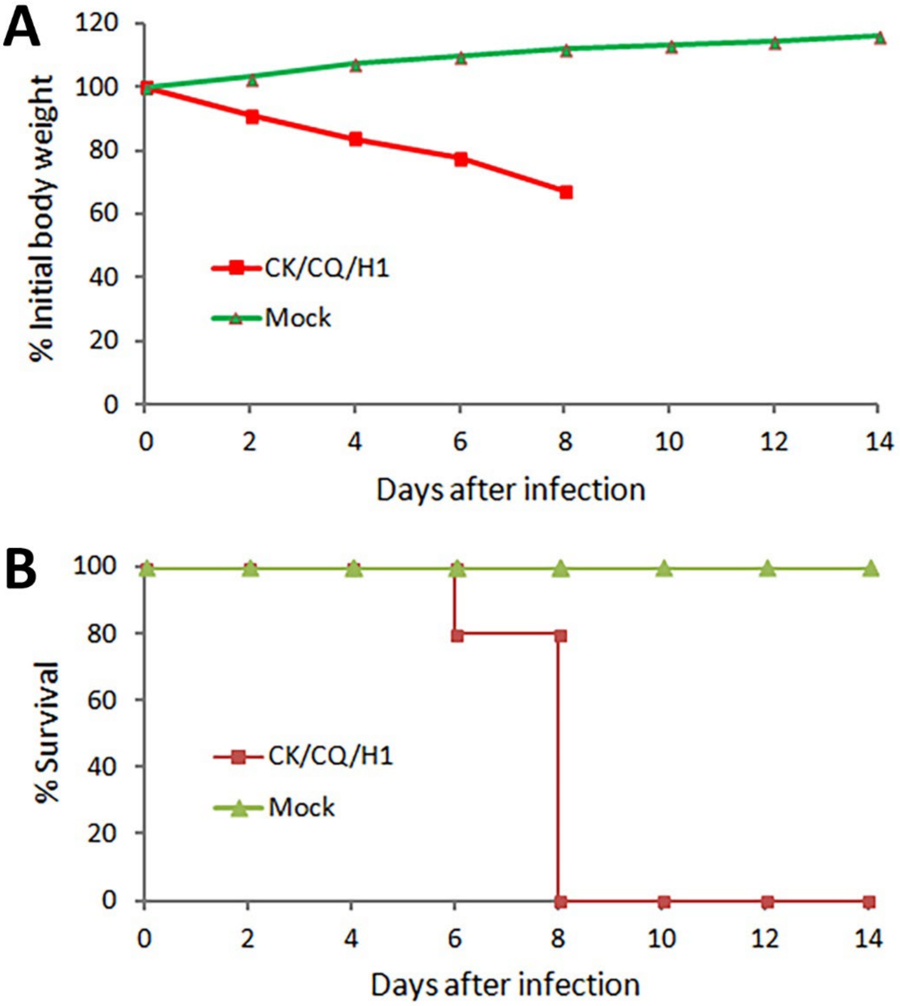

Appendix Figure. Virulence of the novel H5N6 virus in Balb/c mice intranasally inoculated with a 10^6 EID_{50} virus. Bodyweight changes (A) and mortality (B) of BALB/c mice were monitored daily for 14 days.
